# A Combination Therapy for Kawasaki Disease with Severe Complications: a Case Report

**DOI:** 10.1515/med-2020-0002

**Published:** 2019-12-26

**Authors:** Yuriko Abe, Mamoru Ayusawa, Kengo Kawamura, Ryuta Yonezawa, Masataka Kato, Akiko Komori, Ryutaro Kohira, Ichiro Morioka

**Affiliations:** 1 Department of Pediatrics and Child Health, Nihon University School of Medicine, 30-1, Oyaguchi, Kami-cho, Itabashi-ku, Tokyo 173-8610, Japan; 2 Department of Pediatrics, Kobari General Hospital, Noda, Chiba 278-8501, Japan; 3 Department of Pediatrics, IMS Fujimi General Hospital, Fujimi, Saitama 354-0021, Japan; 4 Department of Pediatrics, Tokyo Metropolitan Hiroo Hospital, Tokyo 150-0013, Japan

**Keywords:** Combination therapy, Intravenous immunoglobulin-resistant Kawasaki disease, Methylprednisolone pulse, Plasma exchange

## Abstract

Kawasaki disease (KD) is a form of acute multisystem vasculitis that presents with various complications, including coronary artery aneurysm. Heart failure and brain damage are rare, but life-threatening complications are associated with KD. Here, we describe a 4-year-old girl who developed intravenous immunoglobulin-resistant KD with both left ventricular failure and acute encephalopathy. On day 8 of the illness, the low left ventricular ejection fraction, mitral regurgitation, and low blood pressure, which required continuous administration of dobutamine, were observed during the treatments for KD, including intravenous immunoglobulin. She also appeared unconscious, where the electroencephalogram showed slow waves of activity in all regions of the brain. The cardiac performance improved after she received plasma exchange for three days. However, her unconsciousness with slow waves of activity on electroencephalogram and fever continued after the plasma exchange. Therefore, she was treated with methylprednisolone pulse, followed by prednisolone, as well as intravenous immunoglobulin. Finally, she recovered without any cardiac or neurological sequelae not only at the time she was discharged, but also throughout the follow-up period. The combination therapy using plasma exchange and methylprednisolone pulse may be a treatment option for severe KD with left ventricular failure and acute encephalopathy complications.

## 1 Introduction

Kawasaki disease (KD) is a form of acute febrile multisystem vasculitis of unknown etiology, which most often affects children younger than 5 years of age [1, 2, 3, 4, 5]. It is known that there are various complications associated with KD, including coronary artery aneurysm, and some complications are life-threatening, such as heart failure [6] and encephalopathy [7, 8].

The use of intravenous immunoglobulin (IVIG, a single dosage of 2 g/kg) and oral aspirin (a dosage of 30 mg/kg/day) are established as first-line therapy for KD with efficacy and safety [9]. However, some are IVIG-resistant. Recently, various treatments have been tested for IVIG-resistant KD [10]. However, the treatments for IVIG-resistant KD, especially life-threatening KD, have not yet been established. Here, we report on a rare case of a girl who developed IVIG-resistant KD with left ventricular failure and acute encephalopathy, who was successfully treated with plasma exchange (PE), methylprednisolone pulse (MP), and additional IVIG. Importantly, our patient recovered without any cardiac or neurological sequelae. Written informed consent was obtained from the parents of the patient for the publication of the data.

## 2 Case Report

**Figure 1:**
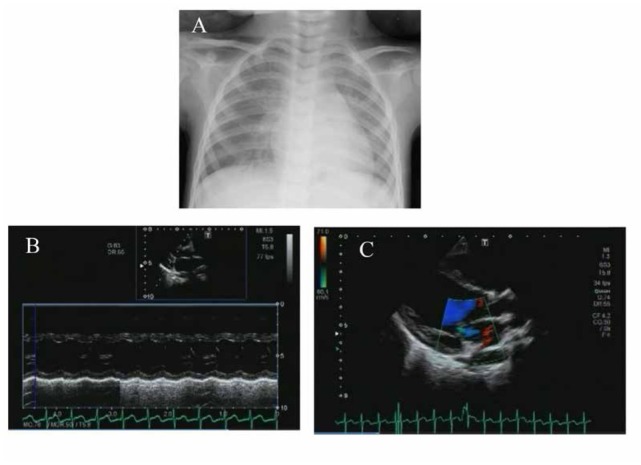
Image examinations on day 8 of illness A: Chest X-ray The cardio-thoracic ratio was 0.53 and congestion was observed in the hilum of both lungs. B and C: Echocardiograms The left ventricular ejection function was 39%, percent fractional shortening was 20.9%, and the left ventricular end-diastolic dimension was 33.9 mm (104% larger than in a normal case).

A 4-year-old girl admitted to our hospital with a highgrade fever, that lasted for seven days, with conjunctival injection, erythema of the lips, cervical lymphadenopathy, and erythema of the palms and soles. The results of her laboratory examinations revealed an increase in white blood cells, in C-reactive protein, in N-terminal pro-brain natriuretic peptide, in interleukin (IL)-6, and in tumor necrosis factor-α (TNF-α) levels (Table 1). Echocardiography showed that the left ventricular ejection fraction was 45% without coronary artery aneurysm. A clinical diagnosis of KD was made, and then the patient was treated with IVIG (a dosage of 2 g/kg), aspirin (a dosage of 30 mg/kg/day), ulinastatin (5000 units/kg/dose, four times a day), and furosemide (1 mg/kg/dose, three times a day) on day 7 of the illness. Despite these treatments, her fever maintained. We found congestion on the chest X-ray scans that were taken on day 8 of the illness (Fig. 1A). In her echocardiography, on day 8 of the illness, the left ventricular ejection fraction was 39% and mild mitral regurgitation was detected, indicating left heart failure (Fig. 1B). Her blood pressure decreased to 80/40 mmHg, which thus required the continuous administration of dobutamine (2 μg/kg/min). The electrocardiogram showed low voltage in the limb leads. In addition, at the same time, unconscious (Glasgow coma scale: 8 [Eye opening: 2, Verbal response: 2, Motor response: 4]) appeared. The electroencephalogram (EEG) showed slow waves of activity in all regions of the brain, especially in the occipital lobe (Fig. 2A), although magnetic resonance imaging (MRI) scans of the brain were normal. Based on these findings, she was diagnosed with acute encephalopathy. As the progression of KD was getting worse, PE was started on day 8 of the illness and was repeated for three days (Fig. 3). The exchange volume was about 1- to 1.5-fold of the circulating plasma volume, which was calculated using the following formula: circulating plasma volume (ml) = body weight (g)/13 × [1-hematocrit (%)/100]. The replacement solution contained 5% albumin. During PE, she was sedated and her breathing was regulated by a respiratory assist device. Her fever went down and the left ventricular function got better during the treatment. However, the fever reoccurred soon after the PE ended. The EEG still showed slow waves of activity at the occipital lobe and parietal lobe. She was treated with MP (30 mg/kg/day) for three days followed by prednisolone administration, as well as IVIG (2 g/kg) to manage the encephalopathy and refractory fever. On day 13 of the illness, her body temperature normalized, and she regained consciousness after discontinuing the sedation and artificial respiratory support. On day 18 of the illness, the EEG showed improvement of slow waves (Fig. 2B), and on day 19, she was able to walk. She was discharged without any cardiac or neurological sequelae. She remained healthy without cardiac or neurological abnormalities during the one year of her follow-up appointments.

**Figure 3:**
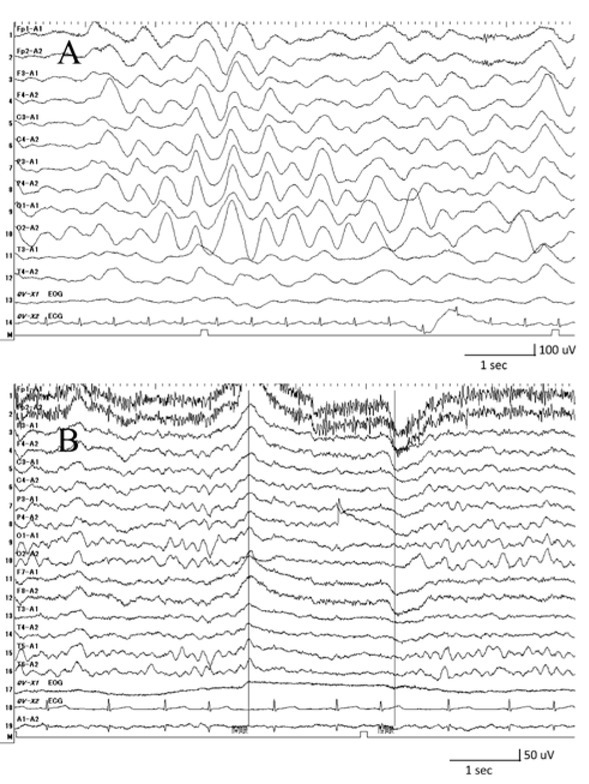
Electroencephalograms A: Slow waves of activity in all brain regions, especially in the occipital lobe, were observed on day 8 of illness B: Abnormal waves were recovered on day 18 of illness.

**Figure 3:**
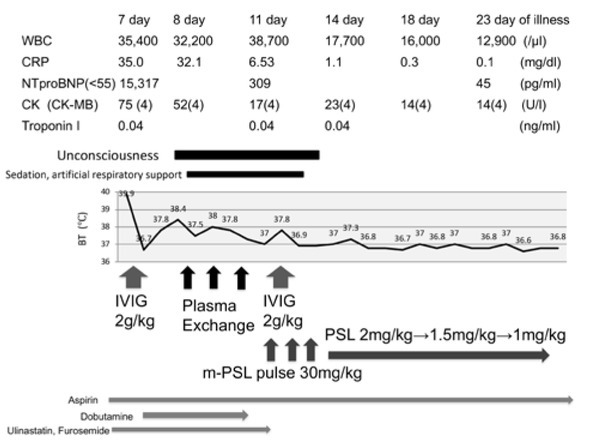
Clinical course Treatments with IVIG (2 g/kg), aspirin (30 mg/kg/day), ulinastatin (5000 units/kg/dose, four times a day), and furosemide (1 mg/kg/dose, three times a day) were started on day 7 of illness. A continuous administration of dobutamine (2 μg/kg/min) was required, because of her low blood pressure. PE therapy was started on day 8 of illness because of left ventricular failure and acute encephalopathy. Due to the recurrence of the fever and EEG abnormalities, MP and IVIG were administered from day 11 of illness. Due to the combination therapy of IVIG, PE, and MP the symptoms of the patient improved. BT: body temperature, CK: creatine kinase, CRP: C-reactive protein, EEG: electroencephalogram, IVIG: intravenous immunoglobulin, KD: Kawasaki disease, MP: methylpredonine pulse therapy, NT-proBNP: N-terminal pro-brain natriuretic peptide, PE: plasma exchange, WBC: white blood cell.

**Table 1:** Laboratory tests on 7 days of illness

WBC	35,400	/μl	CRP	35.0	mg/dl
Neutrophil	93.5	%	Na	134	mEq/l
Lymphocytes	3.0	%	T-bil	0.28	mg/dl
Erythrocyte	436×10^4^	/μl	AST	42	U/l
Hemoglobin	12.4	g/dl	ALT	53	U/l
Hematocrit	37.7	%	CK	75	U/l
Platelet	39.2×10^4^	/μl	CK-MB	4	U/l
			BUN	16.7	mg/dl
			Cr	0.3	mg/dl
			HDL-C	14	mg/dl
			TP	5.9	g/dl
			Alb	1.9	g/dl
			NTproBNP	15,317	pg/ml
			Troponin I	0.04	ng/ml
			IL-6	354 (normal level: <8)	pg/ml
			IL-10	>8 (normal level: <8)	pg/ml
			TNF-α	3.9 (normal level: <2.8)	pg/ml

ALT: alanine transaminase, Alb: albumin, AST: aspartate transaminase, BUN: blood urinary nitrogen, CRP: C-reactive protein, CK: creatine kinase, Cr: creatinine, HDL-C: high density lipoprotein cholesterol, IL-6: interleukin-6, IL-10: interleukin-10, NTproBNP: N-terminal pro brain natriuretic peptide, Na: sodium, T-Bil: Total bilirubin, TNF-α: tumor necrosis factor-α, TP: total protein, WBC: white blood cell.

The research related to human use has been complied with all the relevant national regulations, institutional policies and in accordance the tenets of the Helsinki Declaration, and has been approved by the authors' institutional review board or equivalent committee.

## 3 Discussion

This is a rare case with severe complications that include both left ventricular failure and acute encephalopathy, which was treated with the combination therapy of IVIG, PE, and MP. Myocarditis frequently occurs in the acute phase of KD, which is usually transient; however, there are a few cases of severe myocarditis, which can result in death [11, 12]. In our rare case, the left ventricular failure was serious as there was a need for emergency PE. In regards to neurological complications, the central nervous system is also affected in 0.4% of KD cases. Complications of the central nervous system include seizures, ataxia, cerebral infarction, and subdual effusion [13, 14]. Also, clinical mild encephalitis/encephalopathy with a reversible splenial lesion diagnosed by MRI scans has been reported [15]. However, the finding was not documented on MRI scans in our case. KD with both heart failure and encephalopathy is extremely rare [16], but they are life-threatening complications.

Many previous studies have already reported the efficacy of IVIG in the acute phase of KD to reduce coronary artery abnormalities [9, 10, 17]. However, IVIG-resistant KD is still a serious condition. A recent study has shown that IVIG plus MP prevented coronary artery abnormalities in patients with IVIG-resistant KD [18]. MP might be a good treatment option to prevent coronary artery lesions in KD patients who are unresponsive to additional IVIG [18, 19]. On the other hand, a report has documented that PE was useful to prevent coronary artery abnormalities in patients with severe KD [20]. The removal of inflammatory cytokines might be effective in refractory KD [21]. PE has been used in Japan for over 15 years to prevent coronary artery aneurysm in IVIG-resistant KD patients. However, the efficacy of the combination therapy of PE and MP for severe IVIG-resistant KD patients still does not give any conclusive results. In our case, we initiated PE soon after IVIG treatment, because PE would be more effective in the early disease stage. As expected, PE led a positive effect on our patients’ heart failure. However, unconsciousness and fever were sustained unexpectedly. We, therefore, tried to the following MP for the encephalopathy. Some reports have shown that infliximab, an anti- TNF-α monoclonal antibody, or infliximab plus PE was effective for IVIG-resistant KD [10, 22]. The infliximab was not used in our case, because it is not recommended when the patient has severe heart failure. We speculate that the combination therapy of PE and MP might be safe and prevent cardiac or neurological sequelae in our IVIG-resistant KD.

KD with encephalitis or encephalopathy is also rare. An effective treatment has not been established yet. Some studies have shown that serum IL-6, IL-10, and TNF-α levels were elevated in KD, and these cytokines could cause brain damage in acute encephalopathy following prolonged febrile seizures [23]. Another study has highlighted that the central nervous system complications associated with KD might be due to focal impairment of blood flow caused by cerebral vasculitis [15]. In our case, high levels of serum IL-6 and TNF-α were considered to be the cause of the encephalopathy; regrettably however, the blood flow was not examined.

Another point of discussion is whether MP-alone treatment immediately after initial IVIG treatment followed by aspirin plus prednisolone was sufficiently effective. Suga et al. have reported that the MP combined with IVIG treatments for a KD patient with myocarditis and encephalopathy might inhibit the formation of aneurysms in coronary arteries [16]. However, our patient was a more severe case compared to the subjects of Suga *et al*. study, as the Glasgow coma scale and blood C-reactive protein levels of our patient were extremely high. Matsui *et al*. have reported a case of KD that was resistant to PE, but finally, the patient responded to simultaneous MP and PE [24]. Unfortunately, they did not describe the conscious state or the cardiac function of the patient [24]. As the number of reports on this subject matter is limited, it cannot draw the conclusion that MP-alone treatment is best for heart failure as well as encephalopathy. At least, PE would be a reliable treatment to prevent coronary artery complications.

The combination therapy of PE followed by MP cannot be applied for all severe KD patients, because our report is a case study. However, it may be a treatment option to improve IVIG-resistant KD complicated with left ventricular failure and acute encephalopathy.

## Lists of abbreviations

EEG: electroencephalogram
IL: interleukin
IVIG: intravenous immunoglobulin
KD: Kawasaki disease
MP: methylprednisolone pulse
MRI: magnetic resonance imaging
PE: plasma exchange
TNF-α: tumor necrosis factor-α
